# Unlocking HDR-mediated nucleotide editing by identifying high-efficiency target sites using machine learning

**DOI:** 10.1038/s41598-019-39142-0

**Published:** 2019-02-26

**Authors:** Aidan R. O’Brien, Laurence O. W. Wilson, Gaetan Burgio, Denis C. Bauer

**Affiliations:** 1 CSIRO, Sydney, NSW Australia; 2 John Curtin School of Medical Research, Australian National University, Canberra, ACT Australia

## Abstract

Editing individual nucleotides is a crucial component for validating genomic disease association. It is currently hampered by CRISPR-Cas-mediated “base editing” being limited to certain nucleotide changes, and only achievable within a small window around CRISPR-Cas target sites. The more versatile alternative, HDR (homology directed repair), has a 3-fold lower efficiency with known optimization factors being largely immutable in experiments. Here, we investigated the variable efficiency-governing factors on a novel mouse dataset using machine learning. We found the sequence composition of the single-stranded oligodeoxynucleotide (ssODN), i.e. the repair template, to be a governing factor. Furthermore, different regions of the ssODN have variable influence, which reflects the underlying mechanism of the repair process. Our model improves HDR efficiency by 83% compared to traditionally chosen targets. Using our findings, we developed CUNE (Computational Universal Nucleotide Editor), which enables users to identify and design the optimal targeting strategy using traditional base editing or – for-the-first-time – HDR-mediated nucleotide changes.

## Introduction

Precision medicine^1^ has greatly benefited from advancements in genomic technologies, which enable the identification of potentially disease-causing genetic variants for cancer, neurological or immunological disorders^2–4^. While genome-wide association studies (GWAS) identify common markers or implicate haplotypes for complex traits, high throughput sequencing enables pinpointing of the exact base-changes that may drive these traits^5^. However, the function of many of these detected variants are unknown, and with current functional assays being costly and time-consuming, precision medicine is bottlenecked by this step. The CRISPR-Cas (Clustered Regularly Interspaced Short Palindromic Repeats) system, a recently developed versatile gene-editing technology, allows researchers to modify the DNA or RNA of living cells more easily, and hence promises to accelerate knowledge-gain from functional studies.

One of the most efficient methods for modifying single nucleotides using the CRISPR-Cas system is “base editing”. Base editing requires a catalytically inactive Cas9 or a Cas9 nickase mutant. When fused to a cytidine (C) deaminase or adenosine (A) deaminase enzyme, this can convert C⋅G to T⋅A, or A⋅T to G⋅C, respectively^6,7^. Different groups have recently demonstrated CRISPR base editing to achieve efficiencies of 44% to 100% (median = 82%) in mice, rabbits, rats and human embryos^8–11^. The efficiency depends on factors including the surrounding sequence composition, and the position of the point mutation relative to the protospacer adjacent motif (PAM) at the target site. However, base editing is only efficient within a tight parameter space, e.g. only ~5 bases at each CRISPR-Cas binding site can be targeted with high efficiency^12^. While work is underway to extend the “editing window”^13^, this can result in additional problems. For example, larger editing windows can result in more proximal off-targets. That is, alongside the intended base being changed, all (or some) nearby bases of the same letter within the editing window are changed. The occurrence of proximal off-targets and the restricted range of changes (C → T, G → A, A → G, T → C) limits the application of base editing substantially.

A more-versatile approach to inducing point mutations is via the homology directed repair (HDR) pathway. The HDR pathway is one of two main DNA repair pathways present in organisms from prokaryotes to eukaryotes. Generally, HDR accurately repairs Cas9-induced double strand breaks (DSBs) using a homologous DNA template^14,15^, however, including a mutation in a synthetic DNA template enables HDR to introduce precise changes into the target.

However, compared to base-editing, inducing changes with HDR can be inefficient. Firstly, HDR is in direct competition with the error-prone non-homologous end joining (NHEJ) pathway^15,16^. But unlike NHEJ, which is active throughout the cell cycle, HDR is restricted to the late G2 and S phase of the cell cycle^17^. HDR also competes with the microhomology-mediated end joining (MMEJ) pathway, which is active in the S (and early M) phase^18^. Furthermore, HDR can be negatively influenced by somatic or sporadic mutation in genes such as RAD51, BRCA1 or BRCA2^19,20^. This can lead to challenges not only when working on an organism/cell-line with a pre-existing mutation in these genes, but also when targeting these genes with CRISPR-Cas.

Despite these flaws, HDR is currently the most versatile editing solution as it allows researchers to make nearly any change, from single-nucleotide changes, to insertions of thousands of bases^21^. Therefore, the ability to optimize target site choice, in regards to HDR efficiency, can enable researchers to more easily induce a wide range of changes. But although computational tools exist for predicting the efficiency of CRISPR-Cas^22–25^, they collectively ignore the potential repair outcome. This makes them unsuitable for identifying optimal targets when the desired outcome is a specific change. The absence of tools is likely because factors influencing the repair pathway choice and hence the potential for successfully introducing a point mutation remain unknown.

Here, we identified factors that influence Cas9-mediated HDR efficiency using machine learning on a novel fit-for-purpose dataset. From these insights, we built a computational tool that allows researchers to introduce a wider range of mutations than base editing, by harnessing the versatility of HDR. CUNE, Computational Universal Nucleotide Editor, is available as part of our genome editing toolkit, the GT-Scan Suite, at https://gt-scan.csiro.au/cune.

## Results

### A dataset of genome-wide HDR efficiencies

We curated data from a series of experiments aiming to change a specific nucleotide in mice using Cas9-mediated HDR. To achieve this, we used the CRISPR-Cas9 system to induce a DNA double-strand break (DSB) at a chosen position near the desired point mutation. We used a single-stranded oligodeoxynucleotide sequence (ssODN) template (a point mutation flanked by homology arms, homologous to the PAM strand of the target region) to define the mutation.

The dataset, containing 30 samples (unique HDR targets), is curated from 126 experiments targeting a total of 744 mice (Table 1). On average, each sample includes 25 mice. The 30 samples cover 26 genes and 12 chromosomes. Each sample has several independent variables (guide sequence, ssODN sequence and distance of point mutation from PAM) and dependent variables (HDR efficiency, NHEJ efficiency).

**Table 1:** Our dataset includes results from HDR experiments in 744 embryos.

General	Mice	744
	Samples (unique ssODN/gRNA combinations)	30
	Mice per sample (average)	24.8
	Genes	26
	Chromosomes	12
ssODN	Length (average)	167.07
	3′ arm length (average)	81.50
	5′ arm length (average)	84.57
	GC content (average)	52.75%
Efficiency	HDR (median)	0.199
	NHEJ (median)	0.606

This includes 30 ssODN/gRNA combinations (with approximately 25 embryos each). The median HDR and NHEJ efficiency for each ssODN/gRNA combination is 0.199 and 0.606, respectively. The ssODNs have an average length of 167 (with the arm length between 81 and 85 on average).

We calculated the efficiency values for each of the 30 samples from the 25 mice that comprise each sample. We classified mice carrying the desired mutation as having been repaired via HDR, and mice carrying insertions or deletions (indels) as having been repaired by the error-prone NHEJ. We base these classifications on the knowledge that NHEJ generates indels at DSBs, whereas HDR repairs DSBs according to a template (in this case the ssODN)^26^. The efficiency value for each sample is the number of mice with HDR repairs divided by the number of mice. So for a particular sample, if 5 out of 25 mice were repaired with HDR, the HDR efficiency would be 0.2.

**Figure 1:**
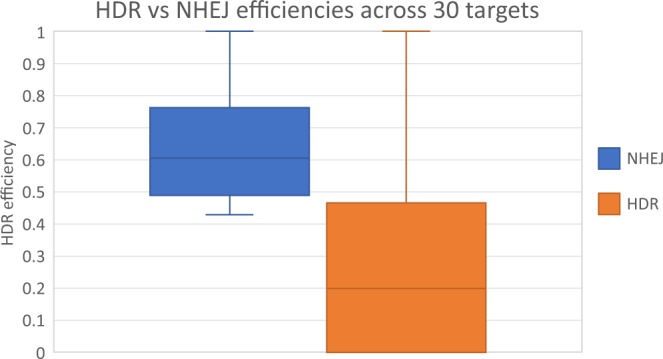
Distributions of HDR and NHEJ efficiencies across the 30 samples. The median HDR efficiency (0.199) is 3-folds lower than the median NHEJ efficiency (0.606). The HDR efficiencies have a higher interquartile range (0.466) than NHEJ (0.334), indicating a larger spread in our HDR efficiencies compared to NHEJ. Despite the larger spread, Q3 of HDR is less than Q1 of NHEJ, indicating the relative inefficiency of HDR versus NHEJ.

The distribution of efficiencies is shown in Fig. 1, clearly demonstrating the 3-fold lower efficiency for HDR (median = 0.199) compared to NHEJ (median = 0.606) when choosing targets traditionally. These values are comparable to previous work^27–29^. We label samples with an HDR efficiency greater than the HDR median (0.199) as high-efficiency, and those less than, as low-efficiency.

### Guide nucleotide composition informs Cas9-mediated HDR insertion rate

Using our dataset, we trained models to predict CRISPR-Cas9-mediated HDR efficiency for new target sites. We took inspiration from computational methods for predicting generic CRISPR-Cas9 activity, which make predominant use of the nucleotide composition of the gRNA^30^. While HDR requires control over the repair-pathway, it may still be predominantly driven by Cas9 activity. We therefore investigated whether the nucleotide composition of the guide is sufficient for predicting knock-in efficiency through HDR.

We used the Random Forest machine learning algorithm to model the data, and the following results are the average across five cross-validated folds. We trained our first model using the guide nucleotide and dinucleotide composition (G1), and subsequent models on other sequence-based features such as repeats or the type of nucleotides present (G2-G4). The nucleotide composition is the count of each nucleotide (i.e. Ts, Gs, etc.) in the sequence, and the dinucleotide composition is the count of adjacent nucleotides (i.e. TTs, GTs, etc.). We recorded metrics including the out-of-bag (OOB) error, area under the receiver operating characteristic (ROC) curve, precision, recall and the number of samples classified correctly. Out of these four models, the simplest model (G1) of the (di)nucleotide compositions produced the lowest error (OOB = 0.25), whereas including the pyrimidine/purine composition (G4) resulted in the poorest model (OOB = 0.33) (Table 2).

**Table 2:** Metrics from five models trained on different decompositions of the guide sequence.

Model	Features	OOB error	ROC	Precision	Recall	Correct
G1	20	0.250	0.84	0.833	0.733	23/30
G2	25	0.258	0.80	0.767	0.6	20/30
G3	29	0.275	0.78	0.787	0.733	22/30
G4	33	0.333	0.75	0.717	0.667	20/30
G5	337	0.617	0.38	0.347	0.4	11/30

Lower is better for OOB error, whereas higher is better for the other values. G1: guide nucleotide composition, G2: G1 + generic repeats, G3: G2 + guide AT/CG composition. G4: G3 + guide pyrimidine/purine composition, G5: local guide nucleotides. Metrics are averaged over the five cross-validated folds.

We also built a model on positional nucleotide information (G5). In contrast to G1, which models the position-independent nucleotide count (i.e. how many Cs are in the sequence), G5 models the presence of each nucleotide at each position (i.e. is there a C present at position 1, position 2, etc.). This position-dependent model performs the poorest of all five, with an OOB of 0.617. The poor performance is likely due to the large number of features (337), relative to the sample size of 30. An increase in this ratio generally correlates with the sparsity of the dataset, i.e. the number of zeros. This phenomenon is known as the “curse of dimensionality” and makes it increasingly difficult for machine learning algorithms to find signal in the data^31^.

### Mutation-to-cut distance does not improve model accuracy

**Figure 2:**
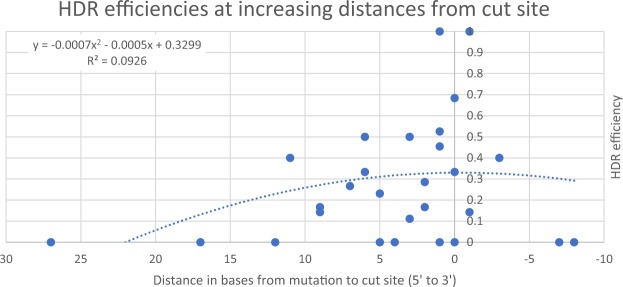
HDR efficiencies for samples (blue circles) plotted against the distance of the mutation from the cut site. Positive domain values are on the 5′ side of the cut on the PAM strand, and negative values on the 3′ side. The average HDR efficiency is highest at distances around 0 bases, and decreases as the distance increases.

With evidence that the distance between the cleavage-site and the mutation has an inverse relationship to HDR efficiency^32,33^, we retrained the above models with distance as an input feature. Supporting previous research, we observed an inverse relationship between distance and HDR efficiency (Fig. 2), albeit with a low coefficient of determination (R² = 0.0926). However, we did not observe an improvement in prediction accuracy of the trained model.

The dataset was designed to capture a wide range of factors, such as gene locus or genomic location influencing HDR efficiency^34^. Relative to those other factors, editing distance seems to be a weak modulator of efficiency as editing may inherently fail at inefficient loci, regardless of distance.

### The 3′ homology arm informs HDR activity

Although we demonstrated that the guide sequence influences Cas9-mediated HDR efficiency, it is likely not influencing HDR directly. The observed higher HDR activity is likely an indirect result of higher DSB frequency. We therefore investigated whether modeling more-direct properties of HDR can improve on our previous best model (G1).

**Figure 3:**
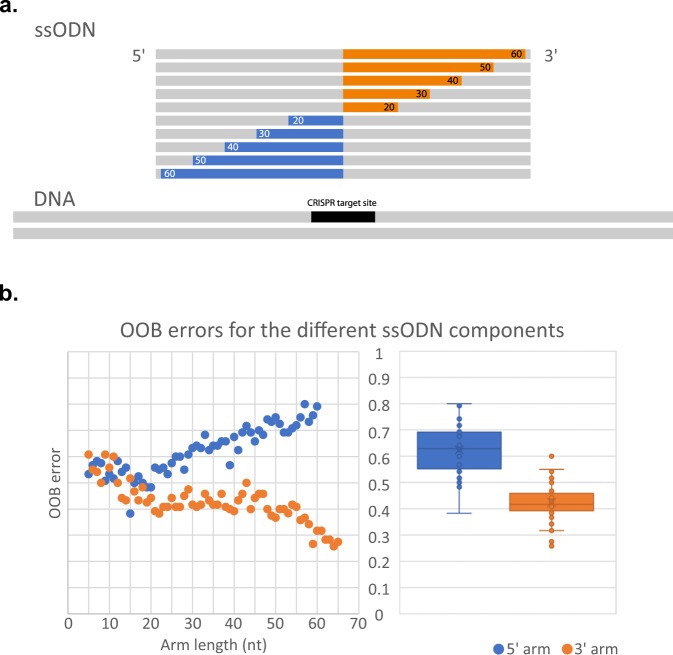
(**a**) The upper bars represent a single ssODN. The blue (5′) and orange (3′) bars represent the regions of the ssODN that we include in each model. The length of this region is displayed on the colored bar. Each region begins at the center of the ssODN and extends outwards. It should be noted that we model regions in increments of 1 base, but only factors of 10 are displayed here for clarity. The lower bar represents the average alignment of the ssODN in regards to the CRISPR target. (**b**) The OOB error for Random Forest models built on the above regions of the ssODN. Regions start from the center and extend outward. Each point represents the error for a model built on one of these regions. The models improve (lower error) as more bases from the 3′ ssODN arm are included. In contrast, the models worsen as more bases from the 5′ ssODN arm are included. The maximum lengths (60nt for the 5′ arm and 65nt for the 3′ arm), are dictated by the smallest respective arm in the dataset. The right plot (sharing the same range) displays the distribution of efficiencies across every ssODN model, comparing the 5′ arm to the 3′ arm.

Due to the key role of the ssODN in inducing the desired point mutation, we hypothesized that a model trained on the ssODN would be able to accurately differentiate between high- and low-efficiency targets. Models trained on the ssODN capture a larger number of nucleotides than the gRNA models (~167 vs 23), see Fig. 3a. However, it appears that this extra information does not contribute to the prediction power, as the ssODN model (O1) performs poorly, with an OOB error of 0.6 (vs. 0.25).

Other groups have investigated the influence of the symmetry and length of ssODN arms on HDR activity, drawing the conclusion that asymmetric ssODNs can improve HDR efficiency^35,36^. The consensus is that a shorter 3′ arm with a longer 5′ arm is the optimal design for efficient HDR, with one theory being that the shorter 3′ arm allows the ssODN to anneal to the DNA target without requiring further processing or strand invasion^36^. Expanding on this, we investigated whether the correlation between HDR efficiency and the ssODN nucleotide composition differs for each arm. We investigated the information content for each arm separately by modeling each arm separately. As presented in Table 3, the 3′ arm (homologous to the PAM/non-target strand) does inform HDR efficiency, with an OOB error of 0.275, while the 5′ arm performs poorly, with an OOB error of 0.792.

**Table 3:** Metrics from three Random Forest models trained on the nucleotide composition of the ssODN. O1 is trained the full ssODN.

Model	Region	OOB error	ROC	Precision	Recall	Correct
O1	Full	0.6	0.54	0.413	0.533	13/30
O2	3′	0.275	0.91	0.803	0.733	22/30
O3	5′	0.792	0.09	0.25	0.267	8/30

O2 is trained on the 3′ arm, and O3 is trained on the 5′ arm (all homologous to the PAM strand).

With a clear difference in predictive power between the two regions of the ssODN (O2 vs O3), we investigated this difference further by modeling different regions of each arm. Each region starts at the middle of the ssODN, next to the point mutation, and extends outward toward the end of the respective arm, one nucleotide at a time (Fig. 3a).

Figure 3b shows that models built on longer regions of the 3′ arm have a better predictive power (60 nucleotides OOB = 0.272) than models built on shorter regions of the 3′ arm (5 nucleotides OOB = 0.608). We observed the opposite trend for the 5′ arm, where model performance worsens with length and the OOB error increases from 0.55 at 5 bases to 0.792 at 60 bases (Fig. 3b). This indicates that the performance improvement is not due to giving additional degrees of freedom to the machine learning model. Adding to the already-established influence of ssODN length^35,36^, we showed that the nucleotide composition of the 3′ arm influences HDR efficiency with symmetric ssODNs.

### Combining the guide and ssODN models

Combining information about the DSB frequency (guide) with information about HDR influencers (ssODN), we built a final model incorporating guide and 3′ ssODN information. We henceforth refer to this model as our mixed model, or M1, see Table 4. M1 successfully identifies the largest number of high-efficiency HDR targets across our cross-validation folds (25 vs 23 (G1) and 22 (O3)). It has the highest recall rate although we observed the same OOB error compared to G1 (0.25).

**Table 4:** Metrics from the mixed model. This Random Forest model includes the 5′ oligo arm (O3) and the guide (G1).

Model	Region	OOB error	ROC	Precision	Recall	Correct
M1	3′ Oligo & Guide	0.250	0.91	0.883	0.8	25/30

CUNE implements M1 to predict HDR efficiency of CRISPR-Cas targets. These results are the average values from 5-fold cross validation.

### Validation

Due to the traditionally low efficiency of HDR-mediated nucleotide edits compared to base editing, it has rarely been used in the literature so far, rendering us unable to find an independent dataset for validation. However, since we continued collecting experimental data that were not included in our test/training dataset, we used these data points for an independent holdout set for validation. We used model M1 to classify the targets in the holdout set and compared the predictions to the truth labels. We observed four out of six low efficiency targets and seven out of nine high-efficiency to be classified correctly (Table 5). This quantifies the accuracy of M1 as 0.733 on unseen data.

**Table 5:** A confusion matrix displaying the classification results of model M1 on our holdout set.

	Predicted: Low	Predicted: High
Actual: Low	4	2
Actual: High	2	7

Out of the 15 targets, M1 classified 11 correctly (7 true positives and 4 true negatives). The prediction accuracy is 0.733.

To quantify the theoretical improvement of choosing HDR targets using our prediction model, as opposed to naively picking targets, we isolated the high efficiency targets from our dataset. This mimics the process a researcher would adopt when using M1 to select optimal targets. We then calculated the average efficiency for these targets and compared this value to the average efficiency of all targets in our dataset, mimicking the process of naively picking sites. This comparison results in an 83% improvement in HDR efficiency for targets chosen using M1 versus targets chosen randomly (with an average efficiency of 0.528 compared to 0.288, respectively).

### Web service for predicting HDR efficiency

We hereby make our predictive model M1 available as a web service: Computational Universal Nucleotide Editor (CUNE). CUNE identifies the optimal way to insert a specific point mutation at a genomic locus. With the efficiency of base editing on average higher than HDR, the service identifies which, if any, base editing system is applicable, using pre-established rules^6,12,37–39^. CUNE also predicts the HDR efficiency for gRNAs and ssODNs around the specified locus.

The base editing component of CUNE is equivalent to BE-Designer^40^, in that both tools identify guides that allow editing of a target using base editing. One difference is that CUNE allows the user to specify the target locus by genomic region, whereas BE-Designer requires the user to enter a target nucleotide sequence. Secondly, while BE-Designer covers a range of PAMs, CUNE specializes in the canonical SpCas9 PAM (NGG), as the most efficient SpCas9 PAM^41,42^. Benchling is another service that provides support for identifying base editing targets^43^. Like BE-Designer, Benchling allows the user to search within a region. But rather than scoring targets based on whether the mutation lies within the editing window, Benchling returns an efficiency score. However, Benchling only supports the classical base editor from Komor *et al*. (2016) and not more-recent base editors like BE4^44^.

In case base editing is not possible, our webpage provides the user with recommendations for high efficiency target sites for inserting point mutations with HDR. These recommendations are ranked by their predicted efficiency, according to our Random Forest model.

## Discussion

We set out to understand the factors that govern HDR-mediated point mutations. We aimed to create a computational tool that makes efficiency-improving recommendations for variables that are easy for the researcher to vary, such as ssODN design and guide. This is especially relevant, as the currently known factors that govern efficiency, such as cell type and locus^34^, are usually fixed parameters for an experiment.

We trained models on different features to investigate how such features influence the HDR efficiency. We chose to use the Random Forest algorithm as it enables us to quantify the contribution of each input feature (variable importance), as well as allowing us to model feature interactions, which provides insights into mechanisms. Random Forests are also resilient to overfitting^45^, which was crucial for our training scenarios that contained more features than samples (e.g. G5).

We hypothesized that the ssODN nucleotide composition would be an influencing factor on HDR efficiency, due to Watson-Crick base pairing between the ssODN and the DNA target being essential for inducing HDR-mediated point mutations. While the nucleotide content of the ssODN 5′ arm was unimportant (O3), the content of the 3′ arm proved to be a major contributor to prediction accuracy (O2).

**Figure 4:**
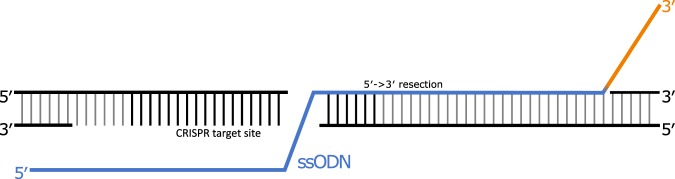
An ssODN (blue/orange) annealed to 5′-3′ resected DNA (PAM strand). ssODNs with regions extending beyond the resected DNA may require further processing or strand-invasion of the DNA target. The sequence composition of this region (orange) has a strong impact on HDR-efficiency.

The importance of the 3′ region is in agreement with the mechanism of HDR. For a cell to proceed with HDR, the 5′ strands at the DSB are degraded^46^. This process, known as 5′ → 3′ resection, results in 3′ overhangs at the DSB (Fig. 4). Therefore, the 3′ region of the ssODN, being complementary with one of the newly-formed 3′ overhangs, is the first region of the ssODN to interact with and bind to the target. We propose that if this occurs, HDR will continue regardless of the 5′ sequence, resulting in the poor predictive performance of our 5′ ssODN models (Fig. 3).

Liang *et al*. observed the optimal length for a 3′ arm to be 30–35 bases, which they base on the 5′ → 3′ resection at the DNA target typically creating overhangs of 30–40 bases^36^. They suggest that arms extending beyond this region are accommodated by further target resecting, 3′ ssODN trimming or strand invasion of the target, while shorter arms can anneal directly to the target. We hypothesized that the efficiency of this process is influenced by nucleotide composition, which we could investigate as our ssODN arms extend beyond the resected region (Fig. 4). In support of the optimal length, we observed our prediction accuracy to temporarily plateau at 20 bases, before continuing to improve at 45 bases, all the way to 60 bases (Fig. 3b orange). This indicates HDR-efficiency is especially sensitive to the nucleotide composition of the region beyond the resected DNA (Fig. 4).

We hypothesized that the distance from the mutation to the PAM would contribute to our model’s accuracy, but this proved not to be the case in our dataset. While we observed the expected inverse correlation between distance and HDR efficiency, reported in previous literature^33,36,47^, the contribution to the computational model was low. It is likely that this is because of the unbalanced nature of this feature in our dataset. For example, Liang *et al*. observed HDR rates of below 5% at distances over 8 bases away from the PAM sequence and rates of 10–30% at, and under, 8 bases away. If we take a distance of 8 bases as the high/low threshold, a balanced dataset would require 50% of the samples to be up to (and including) 8 bases away, with 50% of the samples being over 8 bases away. However, only 6 out of 30 (20%) of our samples are over 8 bases away (Fig. 2), limiting the impact of this feature in our model. This is a form of bias, as we selected gRNA targets based on the proximity of their PAM to the desired mutation. Secondly, especially at short distances, the correlation between the distance and HDR can be quite variable. If we inspect half of our samples, those with the mutation nearest the PAM, we see an average HDR efficiency of 0.33, compared to the overall HDR efficiency of 0.199. Although this is as expected, demonstrating a higher HDR efficiency for samples with the mutation nearer the PAM, these samples have an interquartile range of 0.478. This is nearly half the range of HDR efficiencies observed in the dataset (0 to 1), indicating the high variability, and therefore poor predictive power, of distance.

Our work has resulted in the first computational method for designing efficient experiments for inducing point mutations using base editing and HDR (Supp. Fig. 1). We have provided this as a web service, which will design ssODNs to induce user-specified point mutations. In addition, our web service will also identify base editing targets using pre-existing rules.

## Methods and Materials

### Experiments

#### Ethical statement

All experiments were approved from the Animal Ethics Committee from the Australian National University according to code of practice of the National Health and Medical Research Council (NHMRC) in Australia (AEEC 2014/58 and A2017/44).

#### SgRNA single stranded oligonucleotide design and cloning

Mouse reference genome sequences (GRCm38/mm10 scaffold) were obtained from Ensembl (ensembl.org) or UCSC genome browser (genome.ucsc.edu). SgRNAs were designed to be close to the desired point mutation, with each sgRNA being evaluated for potential off-target effects using online tools such as CRISPOR^24^ or CCTop^25^. SgRNA were designed as a gBlock from IDT (Integrated DNA Technologies, Coralville, IA) encoding a U6 promoter, where the SgRNA scaffold (crRNA and tracrRNA) is the following sequence adapted from Mali *et al*.^48^:

5′-TGTACAAAAAAGCAGGCTTTAAAGGAACCAATTCAGTCGACTGGATCCGGTACCAAGGTCGGGCAGGAAGAGGGCCTATTTCCCATGATTCCTTCATATTTGCATATACGATACAAGGCTGTTAGAGAGATAATTAGAATTAATTTGACTGTAAACACAAAGATATTAGTACAAAATACGTGACGTAGAAAGTAATAATTTCTTGGGTAGTTTGCAGTTTTAAAATTATGTTTTAAAATGGACTATCATATGCTTACCGTAACTTGAAAGTATTTCGATTTCTTGGCTTTATATATCTTGTGGAAAGGACGAAACACCGNNNNNNNNNNNNNNNNNNNGTTTTAGAGCTAGAAATAGCAAGTTAAAATAAGGCTAGTCCGTTATCAACTTGAAAAAGTGGCACCGAGTCGGTGCTTTTTTTCTAGACCCAGCTTTCTTGTACAAAGTTGGCATTA-3′.

The 450 bp gBlock was TA cloned into a pCR2.1-TOPO vector (Thermofisher Scientific, Waltham, MA, USA) according to the manufacturer’s instructions. SgRNAs were also chemically synthesized from IDT (Integrated DNA Technologies, Coralville, IA) as a crRNA and tracrRNA and assembled in a ribonucleoprotein complex with Cas9 enzyme according to the manufacturer’s instructions. 140 bp Ultramer long Oligonucleotides from IDT, at a concentration of 4 nmol, were designed to target the mutation of interest with 70 bp symmetric homology arms. Cas9 recombinant protein was purchased from PNA Bio (Newberry Park, CA, USA).

#### Mouse zygotes microinjection

C57BL/6Ncrl and Swiss Webster CFW/crl mice were obtained from Charles River Laboratories. They were maintained under specific pathogen free conditions under a 12/12-hour light cycle with food and water provided *ad libitum*. Three to five week old C57BL6/Ncrl females were superovulated by 5 µL intraperitoneal injection of Pregnant Mare Serum Gonadotropin (Sigma Aldrich, St-Louis, MI, USA) followed 48 hours later by 5 µL intraperitoneal injection of Human Chorionic Gonadotropin hormone (Sigma Aldrich, St-Louis, MI, USA). Superovulated females were mated with 20 week old stud C57BL/6 N males. For microinjection, 45 to 48 hours following the second hormone injection, zygotes were collected from the oviduct. Pronuclear injections were performed under a DMi8 (Leica, Wetzlar, Germany) inverted microscope apparatus associated with micromanipulators and an Eppendorf FemtoJet microinjection apparatus (Eppendorf, Hamburg, Germany). 50 ng/µL of Cas9 protein was complexed with 3 µM of crRNA and tracrRNA or co-injected with 5 ng/µL of sgRNA plasmid and then mixed with 50 or 100 ng/µL of single stranded oligonucleotides and suspended in UltraPure RNase/DNase-free distilled water (Thermo Fisher Scientific, Waltham, MA, USA) prior to the microinjection. Microinjected zygotes were either surgically transferred into the ampulla of CFW/crl pseudo-pregnant females or cultured overnight at 37 °C in a 5% CO2 incubator and then surgically transferred at 2-cell stage of development.

#### Genotyping

DNA extraction was performed on ear punches from mouse pups over 15 days old, using a crude DNA extraction. The ear punches were briefly lysed in Tris-EDTA/Tween lysis buffer (50mMTris HCl, pH 8.0, 0.125 mM ethylenediaminetetraacetic acid (EDTA), 2% Tween 20) in addition to 1 μL of proteinase K (20 mg/ml in 10 mM Tris chlorate, 0.1 mM EDTA pH 8.0) and incubated at 56 °C for an hour. Subsequently, the DNA was denatured at 95 °C for 10 minutes. Primers were designed from 600–800 bp to amplify the regions encompassing the target sites. PCR was performed using *Taq* polymerase under standard PCR conditions. The PCR products were then purified with ExoSAP-IT (Thermo Fisher Scientific, Waltham, MA, USA) according to the manufacturer’s instructions. Sanger sequencing was performed at the ANU Biomolecular Resource Facility. Specificity of the primers were tested for by using joint amplification of control mouse DNA and Sanger sequencing.

### Data collation

For the results of each CRISPR experiment, we manually collated the data into a spreadsheet. This results in a file with a row for each experiment, where each column stores a variable. We capture the following information:

- Guide sequence
- ssODN sequence
- Observed mutation (arbitrary or point)
- Distance of mutation from PAM

### Preparing the dataset

Here we calculated the efficiency of HDR in inducing a desired point mutation. Generally, there was one experiment (with multiple attempts) aiming to induce a particular mutation. However, some point mutations were the focus of more than one experiment. Therefore, to reduce the potential for “sampling bias”, we merged duplicate experiments into one “sample”. This involved summing up the number of attempts, arbitrary mutations and point mutations, for every attempt at a particular mutation, regardless of which experiment an attempt was part of.

We calculated the HDR efficiency label for each sample by dividing the number of times we observed the desired point mutation for said sample, by the number of attempts to induce a point mutation. Subsequently, we discarded any samples where we observed no mutation (neither HDR nor NHEJ), as our primary focus is influencers of HDR efficiency, not CRISPR-Cas cutting efficiency. This resulted in 30 samples. Each sample has a value from 0 to 1, where 1 indicates 100% HDR, and 0 indicates 0% HDR. For binary classification (high or low HDR), we set a threshold. To result in balanced classes (an equal number of high- and low-efficiency samples), we set the threshold to the median HDR efficiency value, 0.199 (Table 1).

We used Python 3.5 with various packages including scikit-learn, Pandas, NumPy and SciPy. Using Pandas, we can read the data directly from the Excel file into a DataFrame. Here we encode values as integers for compatibility with the scikit-learn Random Forest library. We perform “one-hot encoding” on the discrete non-binary variables. That is, for each unique value in a column, we create new columns where the value is either 1 or 0. For example, the column that stores the first nucleotide from the PAM, “N_1”, is transformed to four columns, “N_1_A”, “N_1_T”, “N_1_C”, “N_1_G”, where each column represents a specific nucleotide at that position.

### Statistical analysis

The primary metric we use is the out-of-bag (OOB) error. This metric takes advantage of one of the properties of the Random Forest algorithm, bootstrap aggregating (bagging). With bagging, each tree is trained on only a subset of samples. Therefore, each tree can be tested on the unseen samples to that tree. This is repeated for every tree throughout the training process. Finally, the average of the errors for each tree results in the OOB.

For further robustness, we partition our dataset using cross-validation^49^ to evaluate our models. Using 5-fold cross validation, we split our dataset into five “folds”. We then train a model on four out of five folds, and test it on the fifth, repeated five times. This allows us to evaluate the prediction error with better generalization to novel data than a train/test set. We use “StratifiedKFold”^50^ to create the folds, as it preserves the distribution of positive and negative samples.

We visualized the performance of each fold using receiver operating characteristic (ROC) curves (Supp. Figs 2–6). These plot the true positive rate against the false positive rate. The average area under the ROC curve for each model is presented in the corresponding tables.

### Scoring the model

The OOB error is the average of error values across each tree in the Random Forest. Because each tree is built using bootstrap aggregating (bagging), different sets of samples remain unseen to each tree. Therefore, the prediction error for each tree can be calculated on the unseen samples to that tree. The mean of these errors is the OOB error, where 0 represents perfect predictions and 1 represents random chance.

With a lack of HDR data in the literature, we validated the model on a more-recent data, completed after training our model. This data includes fifteen samples, being generated in the same way as our training samples (from experiments aiming to induce point mutations in mice). However, these new samples are the result of experiments targeting different genomic loci to those from our original data. We curated these samples in the same way as our original data to generate a dataset of features (guide and oligo nucleotide compositions) and truth labels (high or low efficiency). We scored this data using our mixed model (M1) and compared the predictions to the truth labels.

## Supplementary information

Supplementary material

Dataset 1

## Data Availability

Our source code is available in Supplementary Item 1 and at: https://github.com/BauerLab/GT-scan2-Notebooks. Due to contractual agreements we cannot make our CRISPR targets public, however, our feature matrix is available as Supplementary Dataset 1. Our model trained on this data is publicly available in our GitHub repository.
