# Supplementary material for: Unlocking HDR-mediated nucleotide editing by identifying high-efficiency target sites using machine learning
Source: Sci Rep. 2019 Feb 26;9:2788. doi: 10.1038/s41598-019-39142-0 (PMC6391469; doi:10.1038/s41598-019-39142-0)
Supplement: Supplementary file 1 — Supplementary material [file 41598_2019_39142_MOESM1_ESM.pdf]

Supplementary material for:

"Unlocking HDR-mediated nucleotide editing by identifying high-efficiency target sites using machine learning"

Aidan R. O'Brien, Laurence O.W. Wilson, Gaetan Burgio, Denis C. Bauer

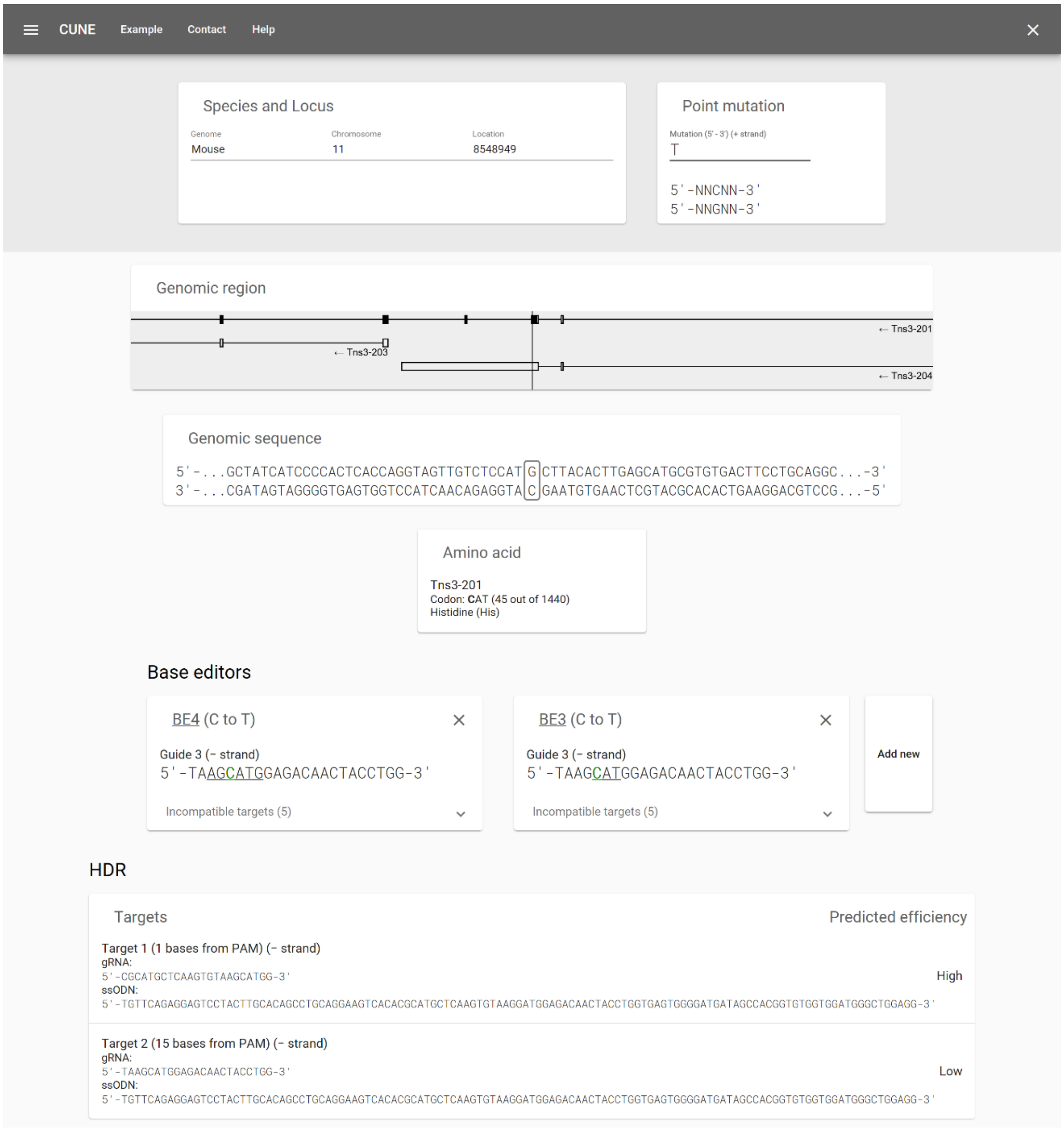

Supplementary Figure 1 – Screenshot of CUNE for an arbitrary region (chr11:8548949) of the mouse genome. Depicted is general information about the specified locus and potential base editor targets. Below the base editors are HDR targets, with the gRNA sequence and ssODN sequence alongside the predicted efficiency.

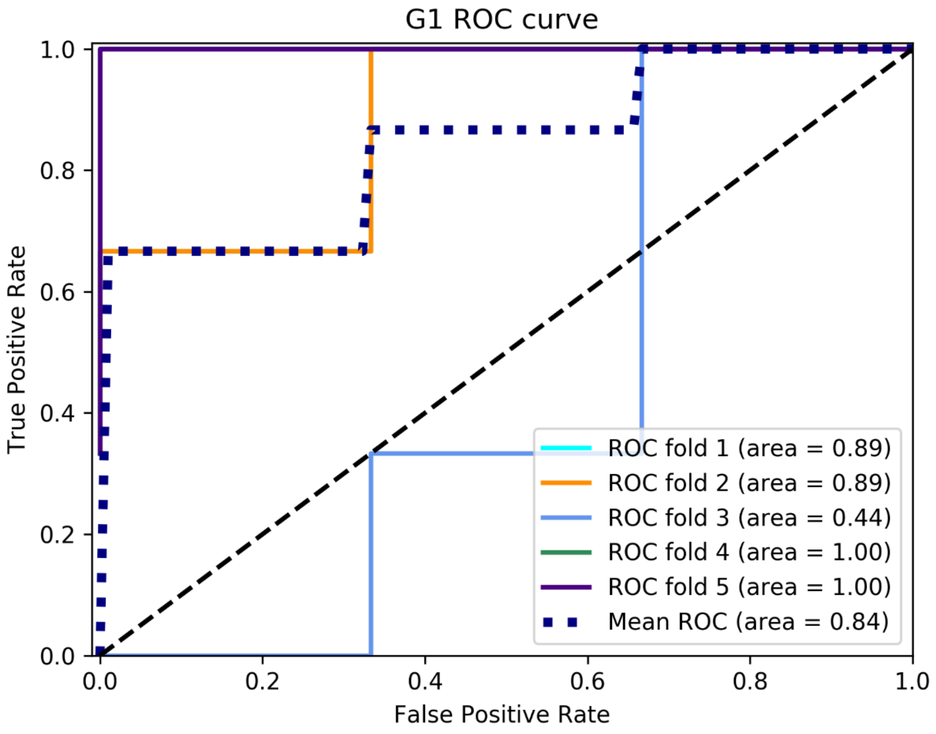

Supplementary figure 2 – ROC curves for the guide (gRNA) model. This models the nucleotide composition of the guide.

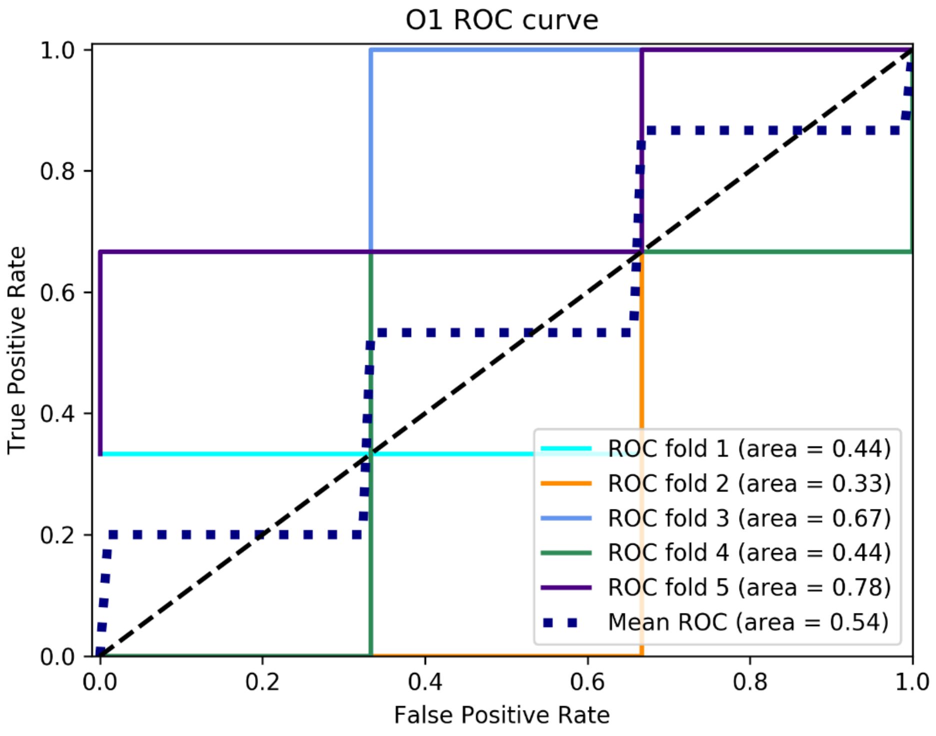

Supplementary figure 3 – ROC curves for the ssODN model. This models the nucleotide composition of the entire ssODN.

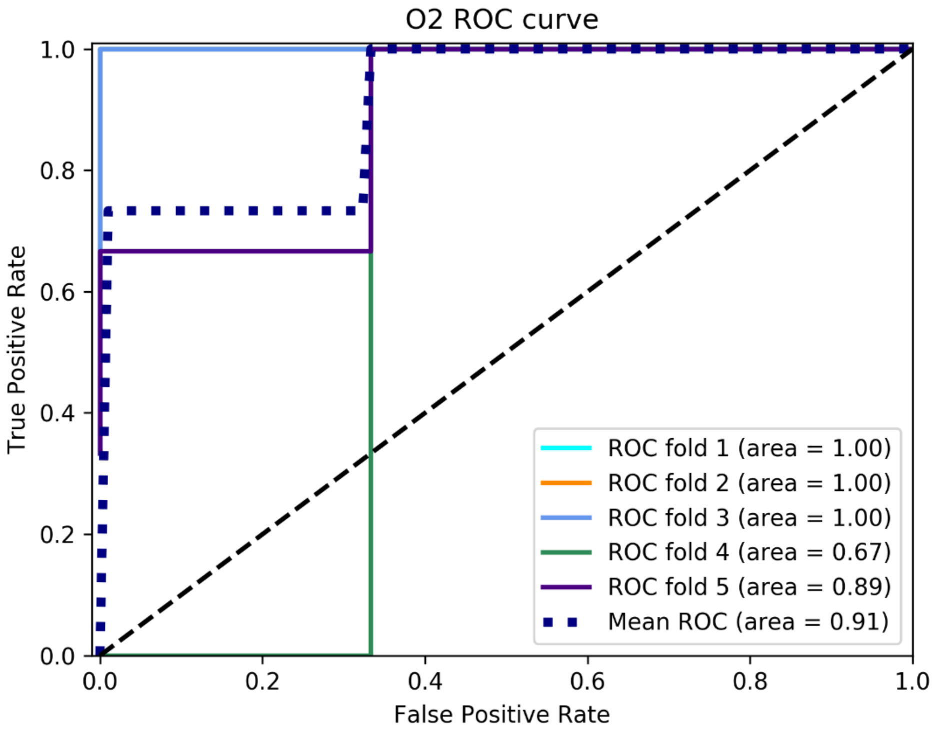

Supplementary figure 4 – ROC curves for the right (3') arm ssODN model. This models the nucleotide composition of the region on the 3' side of the mutation.

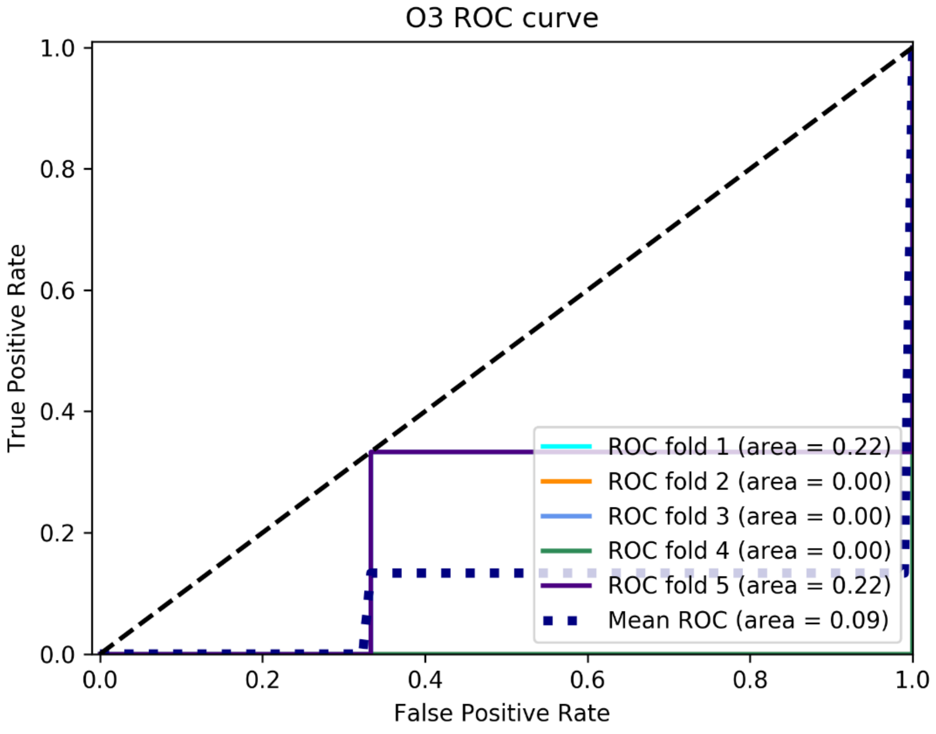

Supplementary figure 5 – ROC curves for the left (5') arm ssODN model. This models the nucleotide composition of the region on the 5' side of the mutation.

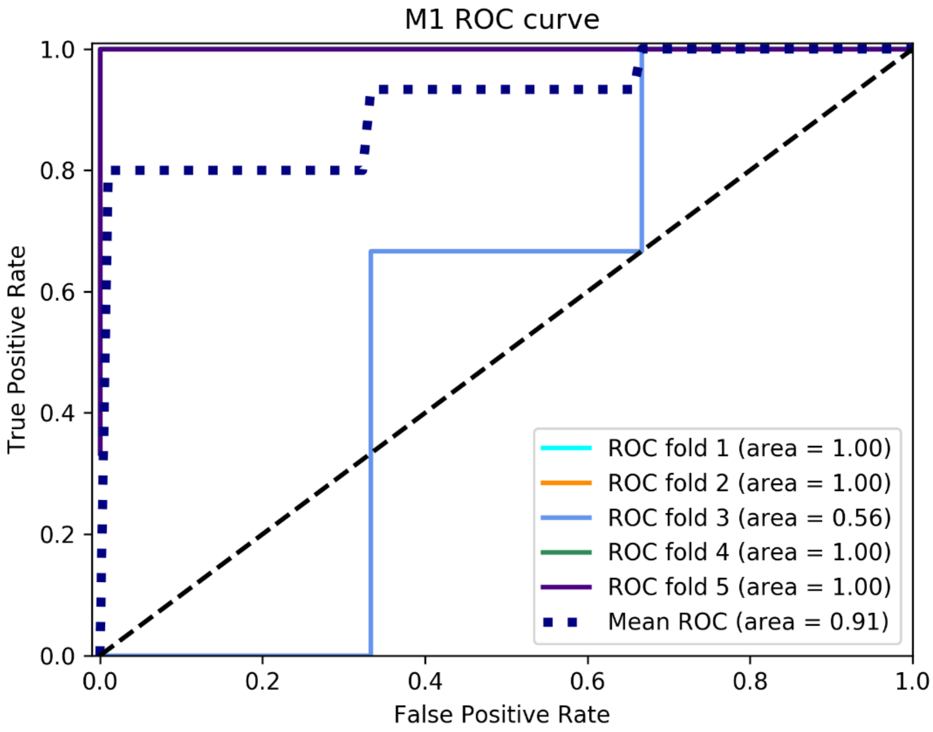

Supplementary figure 6 – ROC plot for the mixed (right arm and guide) model. This models the nucleotide composition of the 3' arm and the gRNA.

HDR modeller

October 9, 2018

0.1 Imports

In [1]:
```
import numpy as np
import pandas as pd
import re

from sklearn.ensemble import RandomForestClassifier
from sklearn.utils import compute_class_weight
```

0.2 Constants

In [2]:
```
LENGTH_OF_PAM = 3
rev = {'A':'T', 'T':'A', 'C':'G', 'G':'C', 'a':'t', 't':'a', 'c':'g', 'g':'c'}
nucleotides = ['A', 'T', 'C', 'G']
dinucleotides = [i + j for i in nucleotides for j in nucleotides]
allnucleotides = nucleotides + dinucleotides
TOKENS_SI = [(1, i) for i in nucleotides]
TOKENS_DI = [(2, i) for i in dinucleotides]
TOKENS = TOKENS_SI + TOKENS_DI

CLASS_LABEL = 'hdr_0.2'
RANDOM_STATE = 3463767
N_TREES = 2000
```

0.3 Functions

In [3]:
```
def read_data(file_name):
    df = pd.read_csv(file_name)
    print(f'Read {len(df)} targets from "{file_name}"')
    return df


## Remove owasites with below the specified cleavage threshold
def remove_unwanted(df, threshold):
    return df[ (df.indels / df.trials) > threshold]


## Returns the reverse complement to a DNA sequence
```

```python
def reverse_complement(sequence):
    return ''.join([rev[i] for i in sequence[::-1]])


## Breaks an oligo down into it's arms (at the point mutation)
def get_oligo_components(guide, oligo, distance_from_pam, mutation_size, strand,
                         truncation=0):

    pam_mutation_index = range(20 - distance_from_pam - mutation_size,
                               20 - distance_from_pam)

    guide_regex = ''.join([nucleotide if i not in pam_mutation_index
                           else '[ATCG]' for i, nucleotide in enumerate(guide)])
    match = re.search(guide_regex, oligo, flags=re.IGNORECASE)
    if match:
        dist = match.span()[1] - distance_from_pam - 3
        arms = [oligo[: dist-1].upper(), oligo[dist:].upper()]
    else:
        oligo = reverse_complement(oligo)
        match = re.search(guide_regex, oligo, flags=re.IGNORECASE)
        dist = match.span()[1] - distance_from_pam - 3
        arms = [oligo[: dist-1].upper(), oligo[dist:].upper()]
    if truncation > 0:
        oligo_arm1 = arms[0][-truncation:]
        oligo_arm2 = arms[1][0: truncation]
    else:
        oligo_arm1 = arms[0]
        oligo_arm2 = arms[1]
    return oligo_arm1, oligo_arm2, oligo_arm1 + oligo_arm2


## Get arms from oligo, optionally with a maximum length
def get_truncated_arms(df, truncation):
    return df.apply(
        lambda _: pd.Series(
            get_oligo_components(_.full_guide_sequence, _.plus_oligo,
                                 _.distance_from_pam, _.mutation_size,
                                 _.strand, truncation),
            index=['oligo_arm1', 'oligo_arm2', 'oligo_trimmed']), axis=1
    )


## Ensure all items are in the same orientation (+)
def get_plus_oligo(guide, oligo, distance_from_pam, mutation_size):
    spacer_size = 20
    pam_mutation_index = range(
        spacer_size - distance_from_pam - mutation_size,
        spacer_size - distance_from_pam
```

```
    )
    guide_regex = ''.join([nucleotide if i not in pam_mutation_index
                           else '[ATCG]' for i,
                           nucleotide in enumerate(guide)])
    match = re.search(guide_regex, oligo, flags=re.IGNORECASE)
    if match:
        return oligo
    oligo_rev = reverse_complement(oligo)
    match = re.search(guide_regex, oligo_rev, flags=re.IGNORECASE)
    if match:
        return oligo_rev


## Merge experiments using the same oligo
def merge_duplicates(df, merge_key):
    tmp_df = df.reset_index().groupby(merge_key, group_keys=False).agg(
        {'index': 'first',
         'point_mutations':sum,
         'indels': sum,
         'trials': sum}).set_index('index')
    cols_to_use = df.columns.difference(tmp_df.columns)

    merged_df = pd.concat([df[cols_to_use], tmp_df], axis=1, join='inner')
    return merged_df


## Generate binary labels
def process_labels(df):
    labels_df = df.loc[:, ['indels', 'point_mutations', 'trials']]
    labels_df.loc[:, 'nhej_ratio'] = (labels_df.indels / labels_df.trials)
    for i in [0.4, 0.5, 0.6]:
        labels_df.loc[:, 'nhej_{}'.format(i)] = \
            (labels_df.indels / labels_df.trials) > i
    labels_df.loc[:, 'hdr_ratio'] = (labels_df.point_mutations / labels_df.trials)
    for i in [0.1, 0.2, 0.3, 0.4, 0.5, 0.6]:
        labels_df.loc[:, 'hdr_{}'.format(i)] = \
            (labels_df.point_mutations / labels_df.trials) > i
    labels_df.loc[:, 'hdr_all'] = \
        (labels_df.point_mutations / (labels_df.point_mutations + labels_df.trials))
    for i in [0.1, 0.18, 0.2, 0.3, 0.4, 0.5, 0.6]:
        labels_df.loc[:, 'hdr_all_{}'.format(i)] = (
            labels_df.point_mutations /
            (labels_df.point_mutations + labels_df.trials)
        ) > i
    return labels_df


## Features from guide sequence
```

```
def process_features_guide_spacer(df):
    prefixes = {'si': 1, 'di': 2}
    new_df = pd.DataFrame(data=None, index=df.index)
    for pre in prefixes:
        l = prefixes[pre] - 1
        for i in range(1,21 - l):
            new_df.loc[:, 'guide_{}_{:02d}'.format(pre, i)] = \
                df.full_guide_sequence.str[
                    - LENGTH_OF_PAM - i - l: - LENGTH_OF_PAM - i + 1
                ]
    global_guide_df = new_df.apply(pd.Series.value_counts, axis=1) \
        .reindex(allnucleotides, axis=1).fillna(0).astype(np.int8) / 20 * 100
    return global_guide_df


def process_features(df, truncation = 0):
    df.loc[:, 'plus_oligo'] = np.vectorize(get_plus_oligo) \
        (df.full_guide_sequence, df.ss_oligo, df.distance_from_pam, df.mutation_size)

    guide_df = process_features_guide_spacer(df)

    ## Create df with the oligo components (arms)
    oligo_arms_df = get_truncated_arms(df, truncation)
    ## Initialize empty df for oligo nucleotide count
    oligo_df = pd.DataFrame(data=None, index=df.index)
    ## Add nucleotide counts to above df
    for i in TOKENS:
        oligo_df.loc[:, 'oligo_arm2_{}'.format(i[1])] = \
            oligo_arms_df.oligo_arm2.apply(
                lambda x: (
                    len(
                        re.findall('(?={})'.format(i[1]), x, flags=re.IGNORECASE)
                    ) / (len(x)/i[0]) * 100
                )
            )

    return pd.concat([guide_df, oligo_df], axis=1)
```

0.4 Train random forest model

Read from a CSV file containing these columns: - point_mutations - indels - trials

- mutation_size
- distance_from_pam
- full_guide_sequence
- ss_oligo

```
In [4]: file_name = 'data/train_set.csv'
```

```
    df = read_data(file_name)
    df = merge_duplicates(df, 'ss_oligo')
    df = remove_unwanted(df, 0.4)

    X = process_features(df)
    y = process_labels(df)['hdr_0.2']

    class_weights = dict(
        zip(
            np.unique(y),
            compute_class_weight('balanced', np.unique(y), y)
        )
    )

    forest = RandomForestClassifier(
        n_estimators=N_TREES,
        oob_score=True,
        class_weight = class_weights,
        random_state=RANDOM_STATE
    )

    forest.fit(X, y)
```

```
Read 98 targets from "data/train_set.csv"
```

```
Out[4]: RandomForestClassifier(bootstrap=True,
                    class_weight={False: 1.28125, True: 0.82}, criterion='gini',
                    max_depth=None, max_features='auto', max_leaf_nodes=None,
                    min_impurity_decrease=0.0, min_impurity_split=None,
                    min_samples_leaf=1, min_samples_split=2,
                    min_weight_fraction_leaf=0.0, n_estimators=2000, n_jobs=1,
                    oob_score=True, random_state=3463767, verbose=0,
                    warm_start=False)
```

0.5 Classify targets

Read from a CSV file containing these columns: - mutation_size - distance_from_pam - full_guide_sequence - ss_oligo

```
In [5]: file_name = 'data/test_oligos.txt'

    df = read_data(file_name)
    X = process_features(df)
    forest.predict(X)
```

```
Read 6 targets from "data/test_oligos.txt"
```

```
Out[5]: array([ True,  True,  True, False,  True, False])
```

Supplementary info 1 – An export of the Jupyter notebook used to generate the CUNE Random Forest model. The latest code will be available at https://github.com/BauerLab/GT-scan2-Notebooks.
